# 青少年体内35种抗生素和4种*β*-受体激动剂的测定及暴露现状分析

**DOI:** 10.3724/SP.J.1123.2023.10022

**Published:** 2024-02-08

**Authors:** Xiaojian HU, Zhenhuan LI, Linna XIE, Hui FU, Ying ZHU

**Affiliations:** 中国疾病预防控制中心环境与人群健康重点实验室, 中国疾病预防控制中心环境与健康相关产品安全所, 北京 100021; China CDC Key Laboratory of Environment and Population Health, National Institute of Environmental Health, Chinese Center for Disease Control and Prevention, Beijing 100021, China

**Keywords:** 超高效液相色谱-串联质谱, 抗生素, *β*-受体激动剂, 青少年, 尿液, 暴露分析, ultra performance liquid chromatography-tandem mass spectrometry (UPLC-MS/MS), antibiotics, *β*-receptor agonists, teenagers, urine, exposure analysis

## Abstract

为了解我国青少年体内抗生素和*β*-受体激动剂的暴露水平,本研究采用同位素稀释-高通量全自动固相萃取-超高效液相色谱-串联质谱法测定某中学442名青少年(11~15岁)尿液中的7类共35种抗生素(5种大环内酯类、4种四环素类、10种喹诺酮类、3种*β*-内酰胺类、11种磺胺类、1种喹噁啉类和1种林可酰胺类)和4种*β*-受体激动剂的水平。尿样测定结果采用尿肌酐进行校正,所得到的样品检出率用于统计分析。采用皮尔森卡方检验对目标物检出率与性别、年龄和体重等级间的相关性进行分析。在调整了混杂因素后,采用Logistic回归模型来评估目标物检出率与不同组别间的相关性。分析结果发现,442名青少年中有397名青少年的尿液中检出抗生素或*β*-受体激动剂,总体检出率为89.8%。除喹噁啉类抗生素未被检出外,实验中共有6类27种抗生素和2种*β*-受体激动剂被检出,检出率为0.2%~59.0%;其中强力霉素、土霉素和阿奇霉素的检出率最高,检出率分别为59.0%、56.1%和34.6%;四环素类和大环内酯类抗生素是主要检出的两类抗生素,检出率分别为81.9%和42.3%;优先作为兽用的抗生素检出率最高(85.1%),其次为人用抗生素(41.0%), *β*-受体激动剂的总体检出率为2.7%。统计分析发现,四环素类抗生素在男性青少年尿液中的检出率高于女性,男性检出风险是女性的2.17倍。大环内酯类抗生素在不同年龄组间的检出率存在差异,与11岁青少年组相比,12、13岁青少年组的检出风险更高。实验发现,大环内酯类抗生素的检出率与青少年的肥胖程度呈正相关;在调整了年龄和性别因素后,肥胖青少年尿液中大环内酯类抗生素的检出风险是正常体重者的2.35倍。研究结果表明,青少年普遍暴露于低剂量的抗生素,且食物和环境中的抗生素可能是青少年体内抗生素暴露的主要来源,大环内酯类抗生素的暴露可能与青少年的肥胖风险相关。

## Abstract (en)

Antibiotics are mainly used for disease treatment and prevention, and *β*-receptor agonists are mainly used in the clinical treatment of respiratory diseases. Both types of drugs are also widely used in animal husbandry and aquaculture to promote animal growth and prevent disease. These drugs enter the human body through many routes and cause harm to human health. Teenagers are in a critical period of growth and development, and long-term antibiotic exposure may have adverse effects on their bodies. In this study, 442 teenagers aged 11-15 years were recruited from a middle school to investigate the body burden of various antibiotics and *β*-receptor agonists. The seven categories of antibiotics, including five macrolides, four tetracyclines, 10 quinolones, 11 sulfonamides, three *β*-lactams, one quinoxaline, and one lincosamide, and four *β*-receptor agonists were determined by isotope dilution and solid phase extraction coupled with ultra performance liquid chromatography-tandem mass spectrometry. Analyte levels were corrected using urine creatinine, and detection rates were used for data analysis. Pearson’s chi-squared test was used to analyze the correlations between detection rate and gender, age, or body mass index (BMI). Logistic regression was used to evaluate the correlation between detection rate and different groups after adjusting for confounding factors. The results showed that 397 teenagers had at least one antibiotic or *β*-receptor agonist in their urine, with a total detection rate of 89.8%. A total of 29 antibiotics and *β*-receptor agonists were detected, and the detection rate of each compound ranged from 0.2% to 59.0%. Doxycycline, oxytetracycline, and azithromycin were the top three drugs with the highest detection rates (59.0%, 56.1%, and 34.6%, respectively). Tetracyclines and macrolides were the two antibiotic categories detected most often, with detection rates of 81.9% and 42.3%, respectively. Among the antibiotics investigated, preferred veterinary antibiotics (PVAs) had the highest detection rate (85.1%), followed by human antibiotics (HAs) (41.0%). The overall detection rate of *β*-receptor agonists was 2.7%. Statistical analysis showed that the male was prone to be exposed to tetracycline antibiotics (odds ratio (OR)=2.17). The detection rates of macrolides differed among the different age groups and were higher in those aged 12-13 years than in those aged 11 years. As the BMI of the teenagers increased, the detection rate of macrolides gradually increased. After adjusting for age and gender, teenagers with obesity were found to be 2.35 times more likely to be exposed to macrolides than those with a normal weight. The findings suggest that teenagers are generally exposed to low levels of antibiotics, that food and the environment may be the main sources of antibiotic exposure in teenagers, and that macrolide exposure may be associated with adolescent obesity.

抗生素是一类具有抑菌和杀菌活性的化合物,其被广泛应用于临床治疗、畜牧养殖和农业生产等领域,并发挥了重要作用^[1,2]^。我国作为抗生素生产和消费的第一大国,2020年抗生素产量高达22.3万吨^[3]^。*β*-受体激动剂能够与肾上腺素*β*受体结合,从而激活受体产生肾上腺素样作用,可用于治疗哮喘或慢性阻塞性肺疾病。*β*-受体激动剂还因具有促生长作用,曾广泛应用于增加畜牧养殖过程中的牲畜重量、提高饲料转化率和增加瘦肉率等。随着抗生素和*β*-受体激动剂用量的不断增多,其在不同环境介质及食品中的检出率也不断增高^[4⇓⇓⇓⇓⇓-10]^,这给生态环境和人体健康均带来了较大的风险^[11]^。抗生素和*β*-受体激动剂会通过环境暴露和食物链进入人体,进而造成一系列人体健康危害。此外,抗生素会导致抗性基因和耐药性等问题的产生,其危害影响更加深远^[12]^。*β*-受体激动剂在人体内蓄积到一定量时,会导致人体发生肌肉震颤、头晕、心跳加快等症状。抗生素根据用途分类,可分为人用抗生素(human antibiotics, HAs)、兽用抗生素(veterinary antibiotics, VAs)、优先作为人用抗生素(antibiotics preferred as HA, PHAs)和优先作为兽用抗生素(antibiotics preferred as VA, PVAs)。我国正在逐渐收紧VAs的限量标准及种类,并明确禁止在动物饲料和饮用水中使用*β*-受体激动剂。青少年阶段是人体生长发育的关键时期,长期受到低剂量抗生素和*β*-受体激动剂暴露会对青少年的健康产生不良影响。目前我国关于青少年体内抗生素水平的研究较少,本研究主要针对山东省某地区青少年体内的35种抗生素和4种*β*-受体激动剂进行测定,旨在了解该地区青少年体内抗生素和*β*-受体激动剂的暴露特征,为科学管理抗生素和*β*-受体激动剂的使用、降低人群健康风险等方面提供重要依据。

## 1 实验部分

### 1.1 仪器、材料与试剂

超高效液相色谱(I Class,美国Waters公司)-串联质谱仪(QTRAP 6500,美国AB SCIEX公司); BY-400C型96孔离心机(北京白洋医疗器械有限公司); LW20型振荡水浴锅(北京莱伯泰科仪器股份有限公司); PL2002型电子分析天平(瑞士Mettler Toledo公司); VORTEX-6型涡旋振荡器(海门市其林贝尔仪器制造有限公司); IQ7005型纯水机(美国Millipore公司); EVOLUTE^®^ EXPRESS ABN 96孔固相萃取板、Extrahera^TM^ HV-5000大容量自动化样品制备工作站、TurboVap^®^ 96 Dual全自动氮吹浓缩仪(瑞典Biotage公司)。

甲醇和乙腈(质谱级,德国Merck公司);甲酸、乙酸和乙酸铵(质谱级,美国Thermo Fisher公司); *β*-葡萄糖醛酸酶(葡萄糖醛酸酶活力≥85000 units/mL,含芳基硫酸酯酶活力≤7500 units/mL,美国Sigma公司); 35种抗生素、4种*β*-受体激动剂的标准品及同位素内标(纯度均>95%)购于天津阿尔塔科技有限公司。

### 1.2 研究对象

选择山东省某中学的442名青少年为研究对象,年龄分布为11~15岁;所有研究对象均为该地区的常住居民,由本人及其监护人签署知情同意书,并配合完成生物样本的采集及相应的调查。根据美国疾病预防控制中心(USCDC)标准^[13]^,本研究按照身体质量指数(BMI)百分位数将青少年体重划分成4个等级,分别为体重过轻(BMI<5%)、正常体重(5%≤BMI<85%)、超重(85%≤BMI<95%)和肥胖(≥95%)。本研究已取得中国疾病预防控制中心环境与健康相关产品安全所伦理审查委员会的许可(编号201813),且每位研究对象的个人信息均得到保密。青少年调查对象的人口学特征见表1。

**表1:** 青少年调查对象的人口学特征

Characteristic	*n*(%)^a)^
Gender	
Female	222 (50.2)
Male	220 (49.8)
Age	
11	80 (18.1)
12	112 (25.3)
13	93 (21.0)
14	128 (29.0)
15	29 (6.6)
BMI grade	
Underweight (BMI<5%)	10 (2.3)
Normal weight (5%≤BMI<85%)	291 (65.8)
Overweight (85%≤BMI<95%)	67 (15.2)
Obese (≥95%)	74 (16.7)

a) numbers of respondents (percent in all, %); BMI: body mass index.

### 1.3 尿样采集

采用医用尿杯对研究对象的即时尿进行收集,之后在现场分装成5 mL/管,并置于冻存管中,于4 ℃冰箱暂存,待样品全部采集完成后统一低温转运至-80 ℃冰箱保存。同时在现场用纯水模拟尿样采集过程,设置现场空白。

### 1.4 尿样检测

#### 1.4.1 尿肌酐检测

参考《尿中肌酐分光光度测定方法》(WS/T 97-1996)^[14]^,利用分光光度法对尿肌酐的含量进行测定。

#### 1.4.2 抗生素和*β*-受体激动剂检测

采用同位素稀释-高通量全自动固相萃取-超高效液相色谱-串联质谱法(UPLC-MS/MS)对尿液中的抗生素和*β*-受体激动剂进行同时测定。首先在1 mL尿液样本中加入10 μL内标(0.4 ng/μL)、200 μL乙酸铵缓冲液(1 mol/L, pH 5)和20 μL *β*-葡萄糖醛酸酶,经37 ℃水浴水解12 h后,采用96孔固相萃取板对其进行前处理,获得样本提取液;利用氮吹仪将提取液浓缩至0.2 mL,并采用UPLC-MS/MS进行测定。具体溶液配制方法、样品前处理方法和仪器分析条件参考《基于高通量全自动固相萃取的超高效液相色谱-串联质谱法测定人尿中16种抗生素和4种*β*-受体激动剂》^[15]^;其中,39种目标物及同位素内标的质谱参数见表2,流动相的梯度洗脱程序见表3。磺胺甲噁唑、甲氧苄啶、磺胺林、磺胺氯吡嗪分别以其对应的^13^C同位素为内标,克拉霉素以其^13^C和D代同位素为内标,土霉素以四环素-D_6_为内标,贝西沙星、甲酰基环丙沙星、8-氟代加替沙星、去甲基氧氟沙星以氧氟沙星-D_3_为内标,磺胺对甲氧嘧啶以磺胺甲嘧啶-D_4_为内标,4-(间甲苯氨基)吡啶-3-磺胺以磺胺甲噁唑-^13^C_6_为内标,头孢克洛和氨苄西林以头孢噻呋-D_3_为内标;其余25种目标物均以其对应的D代同位素为内标。

**表2:** 39种目标物及同位素内标的质谱参数

No.	Compound		Precursor ion (*m/z*)	Product ions (*m/z*)	CEs/eV	DP/V
	**Macrolides** (大环内酯类)					
1	azithromycin (阿奇霉素)		749.7	591.3^*^, 573.5	44, 48	60
2	clarithromycin (克拉霉素)		748.6	158.2^*^, 590.3	34, 25	10
3	roxithromycin (罗红霉素)		837.6	679.3^*^, 158.1	30, 43	30
4	tilmicosin (替米考星)		869.6	696.5^*^, 456.0	63, 22	50
5	erythromycin (红霉素)		734.4	158.3^*^, 576.2	36, 34	30
	**Tetracyclines** (四环素类)					
6	chlortetracycline (金霉素)		479.2	444.0^*^, 462.0	26, 27	30
7	tetracycline (四环素)		445.2	154.0^*^, 410.0	34, 27	20
8	doxycycline (强力霉素)		445.2	428.1^*^, 410.1	25, 35	45
9	oxytetracycline (土霉素)		461.1	426.0^*^, 443.1	26, 18	35
	**Quinolones** (喹诺酮类)					
10	ofloxacin (氧氟沙星)		362.2	318.0^*^, 261.0	24, 39	120
11	enrofloxacin (恩诺沙星)		360.2	316.3^*^, 342.0	28, 31	35
12	pefloxacin (培氟沙星)		334.1	233.0^*^, 290.1	32, 26	130
13	difloxacin (二氟沙星)		400.2	356.1^*^, 299.0	25, 35	125
14	lomefloxacin (洛美沙星)		352.2	265.1^*^, 308.1	29, 23	128
15	sarafloxacin (沙拉沙星)		386.1	299.1^*^, 342.0	27, 32	140
16	besifloxacin (贝西沙星)		394.1	377.1^*^, 279.0	23, 42	25
17	formylciprofloxacin (甲酰环丙沙星)		360.2	342.1^*^, 243.1	25, 43	55
18	8-demethoxy-8-fluoro gatifloxacin (8-氟代加替沙星)		364.3	276.9^*^, 320.2	30, 22	85
19	desmethylofloxacin (去甲基氧氟沙星)		348.2	261.1^*^, 303.9	47, 28	90
	**Sulfonamides** (磺胺类)					
20	sulfamethazine (磺胺甲嘧啶)		278.9	186.2^*^, 156.2	31, 31	25
21	sulfamethoxazole (磺胺甲噁唑)		254.1	91.8^*^, 108.1	24, 31	45
22	sulfadiazine (磺胺嘧啶)		251.1	156.1^*^, 92.0	20, 28	63
23	trimethoprim (甲氧苄啶)		291.3	230.1^*^, 261.2	36, 30	50
24	sulfameter (磺胺对甲氧嘧啶)		281.1	155.9^*^, 107.9	24, 33	120
25	sulfaquinoxaline (磺胺喹噁啉)		300.9	156.1^*^, 108.0	22, 32	67
26	sulfachloropyridazine (磺胺氯哒嗪)		284.9	156.0^*^, 92.0	19, 30	30
27	sulfamonomethoxine (磺胺间甲氧嘧啶)		280.9	156.0^*^, 108.0	26, 35	70
28	4-(3'-methylphenyl)amino-3-pyridinesulfonamide		264.1	168.0^*^, 183.1	42, 34	78
	(4-(3'-甲基苯基)氨基-3-吡啶磺酰胺)					
29	sulfalene (磺胺林)		281.1	156.1^*^, 108.0	22, 27	40
30	sulfaclozine (磺胺氯吡嗪)		285.0	108.0^*^, 92.1	32, 30	50
	***β*-Lactams** (*β*-内酰胺类)					
31	ceftiofur (头孢噻呋)		524.0	241.0^*^, 524.0	23, 32	100
32	cefaclor (头孢克洛)		368.0	105.9^*^, 174.1	24, 18	40
33	ampicillin (氨苄西林)		350.2	105.9^*^, 192.0	20, 22	40
	**Quinoxaline** (喹噁啉类)					
34	quinocetone (喹烯酮)		307.0	273.0^*^, 130.9	29, 31	100
	**Lincosamide** (林可酰胺类)					
35	lincomycin (林可霉素)		407.3	126.1^*^, 359.5	34, 26	120
	***β*-Receptor agonists** (*β*-受体激动剂)					
36	terbutaline (特布他林)		226.1	152.1^*^, 107.1	20, 35	70
37	salbutamol (沙丁胺醇)		240.1	148.0^*^, 166.0	19, 17	60
38		ractopamine (莱克多巴胺)	302.3	121.1^*^, 136.0	30, 26	40
39		clenbuterol (克仑特罗)	277.1	203.0^*^, 168.0	20, 34	30
		**ISs**				
40		azithromycin-D_3_(阿奇霉素-D_3_)	752.6	594.4^*^	46	30
41		clarithromycin-^13^C,D_3_(克拉霉素-^13^C,D_3_)	752.4	162.3^*^	32	53
42		roxithromycin-D_7_(罗红霉素-D_7_)	844.7	686.5^*^	33	26
43		tilmicosin-D_3_(替米考星-D_3_)	872.6	696.4^*^	60	70
44		erythromycin-D_6_(红霉素-D_6_)	740.5	582.3^*^	28	70
45		chlortetracycline-D_6_(金霉素-D_6_)	483.2	448.0^*^	29	55
46		tetracycline-D_6_(四环素-D_6_)	451.1	416.1^*^	29	40
47		doxycycline-D_3_(强力霉素-D_3_)	448.3	431.0^*^	26	30
48		ofloxacin-D_3_(氧氟沙星-D_3_)	365.2	261.1^*^	35	30
49		enrofloxacin-D_5_(恩诺沙星-D_5_)	365.3	245.2^*^	36	40
50		pefloxacin-D_3_(培氟沙星-D_3_)	337.2	293.1^*^	27	125
51		difloxacin-D_3_(二氟沙星-D_3_)	403.1	359.2^*^	29	70
52		lomefloxacin-D_5_(洛美沙星-D_5_)	357.2	270.1^*^	34	134
53		sarafloxacin-D_8_(沙拉沙星-D_8_)	394.3	303.0^*^	35	145
54		sulfamethazine-D_4_(磺胺甲嘧啶-D_4_)	283.0	186.1^*^	26	150
55		sulfamethoxazole-^13^C_6_(磺胺甲噁唑-^13^C_6_)	260.1	162.0^*^	25	30
56		sulfadiazine-D_4_(磺胺嘧啶-D_4_)	255.0	160.0^*^	23	30
57		trimethoprim-^13^C_3_(甲氧苄啶-^13^C_3_)	294.1	231.0^*^	28	130
58		sulfaquinoxaline-D_4_(磺胺喹噁啉-D_4_)	305.1	160.1^*^	21	80
59		sulfachloropyridazine-D_4_(磺胺氯哒嗪-D_4_)	288.9	160.1^*^	20	60
60		sulfamonomethoxine-D_4_(磺胺间甲氧嘧啶-D_4_)	285.0	160.0^*^	26	45
61		sulfalene-^13^C_6_(磺胺林-^13^C_6_)	287.0	162.1^*^	23	50
62		sulfaclozine-^13^C_6_(磺胺氯吡嗪-^13^C_6_)	291.0	114.0^*^	28	50
63		ceftiofur-D_3_(头孢噻呋-D_3_)	527.1	244.0^*^	24	110
64		quinocetone-D_5_(喹烯酮-D_5_)	312.1	278.1^*^	29	35
65		lincomycin-D_3_(林可霉素-D_3_)	410.2	129.1^*^	35	127
66		terbutaline-D_9_(特布他林-D_9_)	235.1	153.1^*^	28	25
67		salbutamol-D_3_(沙丁胺醇-D_3_)	243.2	151.0^*^	20	30
68		ractopamine-D_6_(莱克多巴胺-D_6_)	308.3	168.0^*^	21	24
69		clenbuterol-D_9_(克仑特罗-D_9_)	286.1	204.1^*^	22	40

* Quantitative ion; CE: collision energy; DP: declustering potential.

**表3:** 梯度洗脱程序

Time/min	*φ*(A)/%	*φ*(B)/%
Initial	97.0	3.0
0.3	97.0	3.0
0.5	85.0	15.0
1.0	85.0	15.0
7.0	70.0	30.0
9.5	60.0	40.0
11.0	5.0	95.0
13.0	5.0	95.0
13.5	97.0	3.0
16.0	97.0	3.0

A: 0.1% (v/v) formic acid aqueous solution; B: acetonitrile containing 0.1% (v/v) formic acid.

### 1.5 数据分析

将研究对象按性别、年龄和体重等级分组,计算目标物的检出率。采用皮尔森卡方检验对目标物检出率与性别、年龄和体重等级间的相关性进行分析。在调整了混杂因素后,采用Logistic回归模型来评估目标物检出率与不同组别间的相关性,并计算比值比(OR)。所有统计分析均使用统计软件包R(version 4.0.5),概率值(*p*)<0.050定义为具有统计学意义。

## 2 结果与讨论

### 2.1 方法学验证

采用1.4.2节方法对35种抗生素和4种*β*-受体激动剂的检测进行方法学验证。以目标物与内标的质量浓度比为横坐标(*x*)、峰面积之比为纵坐标(*y*),绘制标准曲线。结果表明,39种目标物在0.02~10 ng/mL范围内线性关系良好,且相关系数均>0.997。向空白尿样中加入低浓度(10倍信噪比所对应的浓度)标准品,配制成7个平行的加标样品,采用上述分析方法进行测定。以3倍标准偏差所对应的质量浓度为方法检出限(MDL)、10倍标准偏差所对应的质量浓度为方法定量限(MQL)。39种目标物的MDL和MQL分别为0.01~0.18 ng/mL和0.02~0.60 ng/mL。在实际尿液样品中分别添加低(0.5 ng/mL)、中(2 ng/mL)、高(5 ng/mL)3个水平的39种目标物,并对加标回收率和日内、日间精密度进行考察。结果表明,39种目标物在3个加标水平下的回收率分别为83.2%~120.8%、86.2%~119.6%和86.9%~117.8%,日内精密度和日间精密度分别为2.0%~7.9%(*n*=6)和2.5%~8.9%(*n*=6)。以上实验结果表明,本文所建立的方法能够满足青少年尿液中35种抗生素和4种*β*-受体激动剂的检测要求。

### 2.2 青少年体内抗生素和*β*-受体激动剂的检出率

尿液检测结果以采用尿肌酐校正后的含量(ng/g)来表示,结果如表4所示。442名青少年中有397名青少年的体内至少检出一种抗生素或*β*-受体激动剂;39种目标物中共有29种目标物被检出,检出率为0.2%~59.0%。四环素类、大环内酯类、磺胺类、喹诺酮类抗生素的检出率均大于10%,检出率分别为81.9%、42.3%、21.0%、15.8%;四环素类抗生素中检出率最高的是强力霉素(59.0%),其次是土霉素(56.1%)和四环素(32.8%);大环内酯类抗生素中检出率最高的是阿奇霉素(34.6%);磺胺类抗生素中检出率最高的是甲氧苄啶(12.2%);喹诺酮类抗生素检出率最高的是氧氟沙星(10.0%),其他抗生素的检出率均小于10.0%。替米考星、培氟沙星、洛美沙星、8-氟代加替沙星、磺胺甲嘧啶、磺胺林、头孢噻呋和喹烯酮共8种抗生素在所有样品中均未检出。在7类抗生素中,四环素类抗生素的检出率和检出含量最高,表明四环素类抗生素是青少年体内的首要抗生素种类。Lyu等^[16]^发现,四环素类和喹诺酮类抗生素是我国于土壤中发现的主要抗生素,磺胺类、大环内酯类、四环素类和喹诺酮类抗生素是我国于地表水中发现的主要抗生素。结合本研究中青少年尿样的检测结果可以说明,四环素类、大环内酯类、磺胺类和喹诺酮类抗生素广泛存在于环境中,并且会通过某些途径进入人体。

**表4:** 研究对象中35种抗生素和4种*β*-受体激动剂的检出率和检出含量

Compound	Category	*n*(%)^#^	Contents/(ng/g)				
			50th	75th		90th	95th
**Macrolides**		187 (42.3)	-	0.1		2.2	35.2
Azithromycin	HA	153 (34.6)	-	-		0.5	5.2
Clarithromycin	HA	9 (2.0)	-	-		-	-
Roxithromycin	HA	43 (9.7)	-	-		-	0.5
Tilmicosin	VA	/	-	-		-	-
Erythromycin	PHA	19 (4.3)	-	-		-	-
**Tetracyclines**		362 (81.9)	-	1.8		12.5	31.3
Chlortetracycline	PVA	43 (9.7)	-	-		-	0.2
Tetracycline	PVA	145 (32.8)	-	0.2		4.3	13.9
Doxycycline	PVA	261 (59.0)	0.1	0.1		0.4	0.6
Oxytetracycline	PVA	248 (56.1)	0.1	0.6		5.3	14.2
**Quinolones**		70 (15.8)	-	-		0.2	0.5
Ofloxacin	PVA	44 (10.0)	-	-		-	0.2
Enrofloxacin	VA	7 (1.6)	-	-		-	-
Pefloxacin	PVA	/	-	-	-		-
Difloxacin	VA	2 (0.5)	-	-	-		-
Lomefloxacin	PVA	/	-	-	-		-
Sarafloxacin	VA	7 (1.6)	-	-	-		-
Besifloxacin	PVA	4 (0.9)	-	-	-		-
Formylciprofloxacin	PVA	8 (1.8)	-	-	-		-
8-Demethoxy-8-fluoro gatifloxacin	PVA	/	-	-	-		-
Desmethylofloxacin	PVA	7 (1.6)	-	-	-		-
**Sulfonamides**		93 (21.0)	-	-	0.2		0.4
Sulfamethazine	VA	/	-	-	-		-
Sulfamethoxazole	PVA	11 (2.5)	-	-	-		-
Sulfadiazine	PVA	1 (0.2)	-	-	-		-
Trimethoprim	PVA	54 (12.2)	-	-	-		0.1
Sulfameter	PVA	1 (0.2)	-	-	-		-
Sulfaquinoxaline	VA	1 (0.2)	-	-	-		-
Sulfachloropyridazine	VA	7 (1.6)	-	-	-		-
Sulfamonomethoxine	VA	31 (7.0)	-	-	-		0.1
4-(3'-Methylphenyl)amino-3-pyridinesulfonamide	PVA	1 (0.2)	-	-	-		-
Sulfalene	PVA	/	-	-	-		-
Sulfaclozine	VA	3 (0.7)	-	-	-		-
***β*-Lactams**		13 (2.9)	-	-	-		-
Ceftiofur	VA	/	-	-	-		-
Cefaclor	PHA	2 (0.5)	-	-	-		-
Ampicillin	PHA	11 (2.5)	-	-	-		-
**Quinoxaline**		/	-	-	-		-
Quinocetone	VA	/	-	-	-		-
**Lincosamide**		17 (3.8)	-	-	-		-
Lincomycin	PVA	17 (3.8)	-	-	-		-
***β*-Receptor agonists**		12 (2.7)	-	-	-		-
Terbutaline		5 (1.1)	-	-	-		-
Salbutamol		/	-	-	-		-
Ractopamine		/	-	-	-		-
Clenbuterol		7 (1.6)	-	-	-		-
Total		397 (89.8)	0.8	6.3	46.1		254.5

# Positive detection numbers (detection rate, %); HA: human antibiotic; VA: veterinary antibiotic; PHA: antibiotic preferred as HA; PVA: antibiotic preferred as VA; -: < method detection limit (MDL); /: no data.

*β*-受体激动剂在青少年尿液中的检出率较低,总体检出率为2.7%。442名青少年中仅有5人检出特布他林(1.1%)、7人检出克伦特罗(1.6%);沙丁胺醇和莱克多巴胺在所有研究对象中均未检出。一份关于我国*β*-受体激动剂使用情况的调查研究发现,尽管克伦特罗和莱克多巴胺已被禁止使用,但二者仍在城市污水中被检出,表明我国仍存在此类药物的使用现象^[17]^。结合本研究结果发现,*β*-受体激动剂能通过各种途径进入人体。

对39种目标物进行混合暴露分析可以发现,同时暴露于两种或两种以上目标物的调查对象占总人数的55.6%;其中四环素类和大环内酯类抗生素复合暴露的检出率最高(36.9%)。在同种类抗生素中,强力霉素和土霉素复合暴露的检出率最高(36.4%);在不同种类抗生素中,阿奇霉素和强力霉素复合暴露的检出率最高(19.2%)。

### 2.3 性别、年龄、BMI与抗生素暴露的关系

将研究对象按性别、年龄和BMI等级分组,并进行统计分析,不同组别中各类目标物的检出率如图1所示。按性别分组,男性青少年中39种目标物的总体检出率为92.4%,女性青少年的总体检出率为87.4%;其中,男性青少年体内大环内酯类和四环素类抗生素的检出率均高于女性;由皮尔森卡方检验结果可知,四环素类抗生素的检出率在男女性别间存在显著差异(*p*=0.004)。Ben等^[11]^发现食物中的抗生素是我国人群体内抗生素暴露的主要来源。有研究^[18]^表明,男性青少年对食物的摄入量一般高于女性,由此推测,食品暴露可能是导致不同性别青少年体内抗生素检出率不同的原因之一。将研究对象按年龄分成4组(11、12、13、14~15岁),采用皮尔森卡方检验进行统计分析。结果发现,仅有大环内酯类抗生素的检出率在不同年龄分组间存在统计学意义(*p*=0.010)。将研究对象按肥胖程度分成4组(体重过轻、正常体重、超重和肥胖),并采用皮尔森卡方检验进行统计分析。结果发现,大环内酯类抗生素的检出率在不同肥胖等级分组间存在统计学意义(*p*=0.003);随着肥胖程度的增加,大环内酯类抗生素的检出率也逐渐增加。有研究^[19]^推测,长期、低剂量的抗生素暴露会对人体肠道微生物菌群造成破坏,使得新陈代谢活动低下、脂肪含量增加,从而导致肥胖。本研究也进一步表明,大环内酯类抗生素暴露可能是导致青少年肥胖的一个因素。

**图1:**
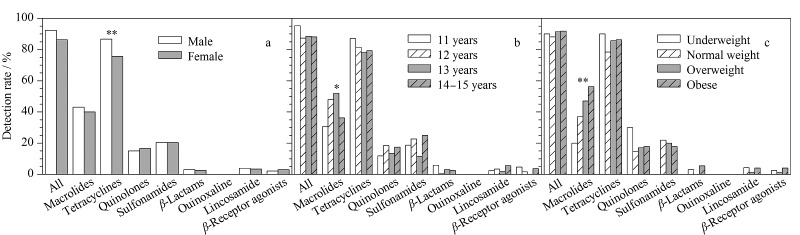
不同(a)性别、(b)年龄和(c)BMI等级分组中抗生素和*β*-受体激动剂的检出率

采用多因素Logistic回归模型对影响大环内酯类和四环素类抗生素检出率的因素进行分析,结果见表5。在调整了年龄和BMI因素后,四环素类抗生素与性别间的相关性依旧存在,在男性青少年中四环素类抗生素的检出风险是女性的2.17倍;在调整了性别和BMI因素后,与11岁组别相比,其他年龄组中大环内酯类抗生素的检出风险更高,其中12、13岁年龄组的检出风险差异具有统计学意义(*p*=0.010和*p*=0.004);在调整了性别和年龄因素后,与正常体重组相比,大环内酯类抗生素在肥胖组中的检出风险最高(OR=2.35, *p*=0.002),且随体重等级的增加而增大。

**表5:** 大环内酯类和四环素类抗生素的多因素Logistic回归分析结果

Influence factor	Macrolides				Tetracyclines	
	OR (95%CI)	*p*			OR (95%CI)	*p*
Gender						
Female (control group)						
Male	0.97 (0.65, 1.47)	0.891		2.17 (1.30, 3.70)		0.004^**^
Age						
11 (control group)						
12	2.23 (1.21, 4.12)	0.010^*^		0.84 (0.37, 1.94)		0.687
13	2.57 (1.36, 4.87)	0.004^**^		0.51 (0.22, 1.16)		0.107
14-15	1.30 (0.71, 2.41)	0.350		0.62 (0.29, 1.32)		0.217
BMI grade						
Normal weight (control group)						
Underweight	0.35 (0.07, 1.70)	0.190		1.72 (0.21, 14.16)		0.613
Overweight	1.49 (0.86, 2.56)	0.155		1.40 (0.67, 2.95)		0.372
Obese	2.35 (1.35, 3.97)	0.002^**^		1.52 (0.70, 3.29)		0.287

OR: odds ratio; CI: confidence interval; * 0.010≤*p*<0.050; ** *p*<0.010.

### 2.4 暴露来源初步分析

人体内抗生素和*β*-受体激动剂的直接来源包括临床用药和自我用药,而其间接来源途径较为复杂,各种介质中的抗生素和*β*-受体激动剂都有可能通过饮食、饮水、呼吸和皮肤接触等途径进入人体。因此,人体内抗生素和*β*-受体激动剂的暴露来源解析难度较大。本研究根据抗生素和*β*-受体激动剂目前的主要用途来初步推断其可能的暴露来源。

按抗生素用途分类,442名青少年中PVAs的检出率最高(85.1%),其次依次为HAs(41.0%)、VAs(12.0%)和PHVs(7.2%)。PVAs如四环素类、磺胺类、喹诺酮类抗生素具有价格低廉和抗菌谱宽等特点,在畜牧养殖中能够加速幼崽生长、缩短饲养周期、增加经济效益。PVAs被广泛应用于畜牧养殖,我国多地外环境或食物中也因此频繁检出此类物质,本研究中检出率最高的强力霉素和土霉素是养殖场广泛使用的四环素类抗生素。还有研究^[20]^发现,叶菜对土壤中的强力霉素具有吸附和富集作用。本研究发现,青少年体内强力霉素和土霉素的检出率最高,但二者在体内的含量却不高(75分位含量分别为0.1 ng/g和0.6 ng/g),由此推断食物暴露可能是青少年体内强力霉素和土霉素的主要来源。阿奇霉素、克拉霉素和罗红霉素仅用作HVs,因此在尿液中检测到的此类抗生素可能主要来源于临床使用或自我用药;但在本研究中,阿奇霉素的检出率高达34.6%,不排除存在阿奇霉素违法用于养殖的可能,已有研究^[21,22]^在鸡肉和鱼肠道内检出阿奇霉素。

*β*-受体激动剂已被禁止用于动物饲养,并被列为安全监督和常规监测的目标物质^[23]^。青少年体内的*β*-受体激动剂主要来源于食物和环境暴露,本研究中*β*-受体激动剂的检出率仅为2.7%,表明我国对*β*-受体激动剂的管控较好,青少年经食物和环境暴露*β*-受体激动剂的风险较小。

## 3 结论

本研究采用同位素稀释-高通量全自动固相萃取-超高效液相色谱-串联质谱法测定了青少年体内35种抗生素和4种*β*-受体激动剂的暴露水平,并初步了解到,青少年普遍受到低浓度抗生素暴露的影响,且不同性别、年龄和BMI等级分组中青少年的暴露风险不同。优先兽用抗生素是青少年体内的主要抗生素种类,而*β*-受体激动剂对青少年的暴露风险较小。本研究也存在不足之处,实验仅针对山东省某地区的青少年展开调查,无法代表全国青少年抗生素和*β*-受体激动剂的整体暴露水平,且本研究属于横断面调查,不能反映抗生素和*β*-受体激动剂长期、低剂量暴露对人体健康的影响。

## References

[1] Moser C, Lerche C J, Thomsen K, et al. APMIS, 2019, 127(5): 361 30983040 10.1111/apm.12951

[2] Mcmanus P S, Stockwell V O, Sundin G W, et al. Annu Rev Phytopathol, 2002, 40: 443 12147767 10.1146/annurev.phyto.40.120301.093927

[3] Cai D M, Ouyang J, Ding J J, et al. Chinese Journal of Analytical Chemistry, 2022, 50(3): 327

[4] Zhou L J, Ying G G, Zhao J L, et al. Environ Pollut, 2011, 159(7): 1877 21501908 10.1016/j.envpol.2011.03.034

[5] Pan M, Chu L M. Sci Total Environ, 2017, 599/600: 500 28482307 10.1016/j.scitotenv.2017.04.214

[6] Chen C, Li J, Chen P, et al. Environ Pollut, 2014, 193: 94 25016103 10.1016/j.envpol.2014.06.005

[7] Shi Y, Gao L, Li W, et al. B Environ Contam Tox, 2012, 89(4): 857 10.1007/s00128-012-0761-1 22875285

[8] Lei K, Zhu Y, Chen W, et al. Environ Int, 2019, 130: 104919 31226562 10.1016/j.envint.2019.104919

[9] Song C, Zhang C, Kamira B, et al. Environ Toxicol Chem, 2017, 36(11): 2899 28585696 10.1002/etc.3876

[10] Fan S, Miao H, Zhao Y F, et al. J Agric Food Chem, 2012, 60(8): 1898 22300587 10.1021/jf2043828

[11] Ben Y, Hu M, Zhong F, et al. Environ Res, 2022, 212(Pt C): 113387 10.1016/j.envres.2022.113387 35513060

[12] Chen X, Yang Y, Ke Y, et al. Sci Total Environ, 2022, 814: 152852 34995606 10.1016/j.scitotenv.2021.152852

[13] Centers for Disease Control and Prevention. Defining Childhood Weight Status.[2023-10-20]. https://www.cdc.gov/obesity/basics/childhooddefining.htmlhttps://www.cdc.gov/obesity/basics/childhooddefining.html

[14] WS/T 97-1996.

[15] Li Z H, Hu X J, Lu Y F, et al. Chinese Journal of Chromatography, 2023, 41(5): 397 37087605 10.3724/SP.J.1123.2022.08025 PMC10122765

[16] Lyu J, Yang L, Zhang L, et al. Environ Pollut, 2020, 266(Pt 1): 115147 32673932 10.1016/j.envpol.2020.115147

[17] Zhong Y, Hou C, Gao X, et al. Sci Total Environ, 2023, 10: 894 10.1016/j.scitotenv.2023.164956 37343858

[18] Ju L H, Zhang Q, Yang Z Y, et al. Chinese Journal of Hygiene Research, 2023, 52(1): 67 36750332 10.19813/j.cnki.weishengyanjiu.2023.01.012

[19] Wang Y Q, Yan C H. Chinese Journal of Applied Clinical Pediatrics, 2020, 35(1): 78

[20] Zhang S X, Luo P P, Bao E D, et al. Jiangsu Journal of Agricultural Sciences, 2018, 34(5): 6

[21] Liu Z Q. [MS Dissertation]. Zhengzhou: Henan Agricultural University, 2017

[22] Ding Q, Wang J F, Feng Y C, et al. Chinese Journal of Food Safety and Quality, 2022, 13(4): 1141

[23] Ministry of Agriculture of the People’s Republic of China. Announcement by the Ministry of Agriculture No.1519. (2011-01-13) [2023-10-20]. http://www.moa.gov.cn/gk/zcjd/201101/t20110113_1806088.htmhttp://www.moa.gov.cn/gk/zcjd/201101/t20110113_1806088.htm

